# The Impact of Career Growth on Knowledge-Based Employee Engagement: The Mediating Role of Affective Commitment and the Moderating Role of Perceived Organizational Support

**DOI:** 10.3389/fpsyg.2022.805208

**Published:** 2022-03-17

**Authors:** Zhu Jia-jun, Song Hua-ming

**Affiliations:** College of Public Administration, Nanjing Agricultural University, Nanjing, China

**Keywords:** knowledge worker, career growth, employee engagement, perceived organizational support, affective commitment

## Abstract

Based on social exchange theory and attribution theory, this paper explores the role of affective commitment and organizational support in the relationship between career growth and the engagement of knowledge workers. The results show that (1) career growth has a positive impact on knowledge workers’ organizational engagement; (2) career goal progress and professional ability development promote job engagement; (3) career growth has a positive effect on affective commitment, which in turn influences employee engagement; (4) affective commitment plays a mediating role in the effect of career growth on engagement; and (5) perceived organizational support positively moderates the relationship between career growth and affective commitment.

## Introduction

In the knowledge economy era, knowledge workers have become an important pillar of enterprises. Their engagement is crucial to the realization of the strategic goals, performance, and vision of enterprises. Employees’ engagement is affected by psychological contract (Kolb et al., 1979). With the continuous change of the external competitive environment of enterprises, the competition between enterprises has become increasingly fierce. A single form of economic contract has been difficult to mobilize the enthusiasm of employees, which is not conducive to the production and creation of employees. Today’s employees pay more attention to their own long-term development, not only focus on material satisfaction, but also pursue psychological needs. Eager to achieve step-by-step promotion through their own ability, so as to obtain a sense of psychological achievement. If this psychology cannot be satisfied, employees’ sense of psychological loss will reduce their sense of dedication and urge them to leave their jobs (Robinson et al., 2004). Knowledge workers attach higher importance to career growth than other employees. In addition to their material pursuit, the organizational atmosphere, the development platform, and other elements are their increasing concerns. When feeling that the organization provides strong support, they may be more attached to the organization psychologically, thus producing affective commitment and enhancing their devotion to work to a certain extent. Therefore, the academic community needs to study how to make the organization-member relationship more stable and make knowledge employees more willing to contribute more to the organization.

Until now, research in the field of engagement has mostly focused on the following topics: (1) exploring the antecedent variables of engagement. Environmental variables such as job characteristics and organizational support can significantly predict engagement (Saks, 2006; Rothmann and Joubert, 2007); In terms of individual factors, such as responsibility has a positive predictive effect on engagement, while neuroticism is negatively correlated with engagement (Schaufeli and Bakker, 2004). The typical characteristics of dedicated employees are high extroversion, low neuroticism and high flexibility (Langelaan et al., 2006). (2) Studying the impact of employee engagement on enterprise production and operation indicators, such as innovation performance and employee retention rate. There is a strong negative correlation between engagement and employee turnover intention, which can positively predict organizational profits and productivity (Harter et al., 2002; Schaufeli and Bakker, 2004). At present, scholars often regard variables such as career growth and employee engagement as single dimensional concepts and establish research models between them. Previous studies have shown that career growth has a positive effect on affective commitment (Meyer and Herscovitch, 2001), confirming the notion that the employees’ perceived organizational support promotes their higher emotional attachment to the organization (Shore and Wayne, 1993; Anitha, 2014). However, the constituent elements of career growth and engagement are multi-dimensional, which can be divided into different dimensions for more detailed discussion. The discussion on the influence path and improvement measures of knowledge worker engagement still needs to be further studied. Another research gap is, as mentioned above, although there is a significant body of literature on employee engagement, prior research primarily focuses on the perspective of the external environment and individual factors, but ignored the important impact of employees’ internal feelings of emotional care and organizational support on employee engagement.

To fill these research gaps, this study explores whether knowledge workers’ career growth can improve employee engagement and determines the specific relationship between dimensions of career growth and employee engagement. The paper also deeply explores the role of affective commitment and organizational support in the influence path of career growth on knowledge workers’ engagement. We aim to provide theoretical guidance and management suggestions for enterprises to better manage knowledge workers with the background of a new economy.

This study contributes to the literature in three aspects. First, in the era of knowledge economy, knowledge workers have become the source of enterprise competitive advantage. This paper enriches the existing literature on knowledge employee engagement by drawing new attention to career growth as an important predictor. Second, we subdivides the career growth and employee engagement to better explores what kind of career growth is more needed by knowledge employees and how to better improve employees’ engagement from the perspective of career objectives, salary promotion and ability growth, which provides new insights for the mechanism of knowledge employees’ engagement. Third, we combine affective commitment and organizational support into our research model and fill the research gap of the relationship between career growth, affective commitment, organizational support and employee engagement.

## Hypotheses Development

### Career Growth and Employee Engagement

Career growth emphasizes the career progress made by employees at a certain point—including not only the current internal growth of the enterprise but also the career growth of individuals in the process of inter-organizational mobility, especially the growth rate of individuals in the enterprise. The level of employee engagement not only means that the employee exhibits behaviors beneficial to the organization within the organization but also includes the individuals’ attitude toward the enterprise and recognition of the value of their existence in the enterprise. Saks (2006) divided engagement into two dimensions: job engagement and organizational engagement, and job engagement is manifested in high work engagement and concentration, organizational dedication is manifested in a strong willingness to stay on the job, and favorable behaviors for organizational production occur in high frequency.

Knowledge workers attach great importance to whether they can get good development opportunities and resources in the enterprise, which to some extent shows that career growth has a significant impact on employees’ work attitudes. Individuals will reciprocate with a high level of engagement when organizations provide employees with opportunities to improve their professional ability (e.g., increasing their salary and promoting them) (Rothmann and Joubert, 2007). The resources invested by enterprises to improve the professional level of employees could promote their work involvement (Fateme et al., 2017). Tang et al. (2015) divided developmental human resource management into three dimensions—career growth, training opportunities, and performance evaluation—believing that all three dimensions can positively affect employee engagement, meet the needs of enterprises and employees, and trigger a benign interaction between employers and employees. Based on this, the following hypotheses are proposed:

H1: Career growth has a positive impact on employee engagement.

H1a: Career goal progress has a positive impact on organizational engagement.

H1b: Professional ability development has a positive impact on organizational engagement.

H1c: Organizational rewards development has a positive impact on organizational engagement.

H1d: Career goal progress has a positive impact on work engagement.

H1e: Professional ability development has a positive impact on work engagement.

H1f: Organizational rewards development has a positive impact on work engagement.

### Career Growth and Affective Commitment

Due to the recognition of organizational values, organizational culture, organizational atmosphere, and behavior mode, individuals have an emotional attachment to their organization, which is affective commitment (Allen and Meyer, 1991). Its special feature is that employees actively connect themselves with the organization, leading to a series of positive emotions and behaviors. One of the sources of affective commitment is the perception of the value of completing the work task or the task itself. Employees infer the degree of commitment of the organization by virtue of their perception of the degree to which the organization attaches importance to their contribution and cares about their growth (Meyer and Herscovitch, 2001).

The level of affective commitment of employees who are highly committed to career growth is significantly higher than that of other employees (Weer and Greenhaus, 2020). After the career growth expectations of knowledge workers are satisfied, they gain a sense of reciprocity, as well as a strong attachment to the organization, and want to keep their identity as members of the organization at the emotional level to promote the generation of their affective commitment. Nouri and Parker (2013) pointed out that there is a social exchange between employees and enterprises and that career development opportunities represent important benefits provided to employees by organizations. Therefore, individuals improve their affective commitment to an organization after obtaining opportunities such as job promotion and growth ability. Personal resources can have an impact on decision-making (Wang et al., 2022). When organizations provide employees with expected opportunities and resources for professional ability development, individuals and enterprises will gradually become better integrated, thus generating a mutual bond and fostering organizational commitment. Bashir et al. (2020) also indirectly confirmed the role of career growth in enhancing organizational commitment when discussing the role of transformational leadership in organizational commitment. The high affective commitment of organization members is affected by their perceived positive support of the organization. Based on this, this paper proposes the following hypotheses:

H2: Career growth has a significant positive impact on affective commitment.

H2a: Career goal progress has a significant positive impact on affective commitment.

H2b: Professional ability development has a significant positive impact on affective commitment.

H2c: Organizational rewards development has a significant positive impact on affective commitment.

### Affective Commitment and Employee Engagement

Affective commitment is a kind of stable psychological binding force that can guide individual behavior to some extent, reflecting the mental state or psychological state of employees who have a sense of obligation, need and desire to maintain membership in the organization to some extent (Allen and Meyer, 1991). While employee engagement, which emphasizes the relationship between work situation, organizational situation and individual psychology, focuses on the process of employees’ individual psychological experience of these two situations and the degree of self-expression and employment in the specific process of affecting job performance (Anitha, 2014). Affective commitment can promote employees to keep consistent with the original behavior direction when the expected fair return or income is not realized (Lalwani and Sharon, 2013).

Commitment is closely related to work involvement. Employees with high commitment to the organization also tend to hold a high level of engagement (Judge et al., 2001). When knowledge workers make a strong level of affective commitment to the enterprise, they have a stronger recognition of the strategic goals and code of conduct of the enterprise and are willing to make more efforts for the construction of the organization with their knowledge, skills, and accomplishment, all of which improve employee engagement in different aspects. High commitment to the organization makes them willing to work hard for the organization, even sacrifice their spare time, and strongly hope to maintain an exchange relationship with the organization (Mercurio, 2015). However, individuals with low affective commitment tend to consider gains and losses from the self-interest perspective and, unable to maintain a high degree of engagement, are ready to withdraw from the organization at any time (Cao et al., 2019). Accordingly, we propose:

H3: Affective commitment has a positive impact on employee engagement.

H3a: Affective commitment has a positive impact on organizational engagement.

H3b: Affective commitment has a positive impact on work engagement.

### The Mediating Role of Affective Commitment

Affective commitment is a bridge between career growth and knowledge worker engagement. Managers can promote employees’ job engagement by ensuring their access to resources and support and improving their affective commitment level (Wayne et al., 2002). The career growth obtained by employees reflects the attitude recognized by the organization, which means the satisfaction of personal development needs and the realization of self-value. After obtaining professional growth, employees will make affective commitment to the enterprise through the principle of reciprocity, and will return to the organization with the commitment of loyalty, staying in the organization and abiding by the organization’s norms, which will lead to more positive feelings and attitudes to work. For example, the realization of employees’ career goals in organizations and the increase of career-related knowledge and skills will have a positive impact on employee engagement through organizational commitment (Weng et al., 2010). If enterprises construct employees’ promotion and personal growth from the perspective of strategic management, the level of affective commitment of individuals to the organization will be further enhanced, which in turn, will improve the frequency of positive behaviors of employees to the organization (Li, 2020). Based on this, this paper proposes the following hypotheses:

H4: Affective commitment plays a mediating role in the relationship between career growth and employee engagement.

H4a: Affective commitment mediates the relationship between career goal progress and organizational engagement.

H4b: Affective commitment mediates the relationship between career goal progress and work engagement.

H4c: Affective commitment mediates the relationship between professional ability development and organizational engagement.

H4d: Affective commitment mediates the relationship between professional ability development and work engagement.

H4e: Affective commitment mediates the relationship between organizational rewards development and organizational engagement.

H4f: Affective commitment mediates the relationship between organizational rewards development and work engagement.

### The Moderating Role of Perceived Organizational Support

Perceived organizational support by employees has a strong positive effect on organizational commitment (Li, 2020). Individuals who feel supported by their organizations are happy, protected, and validated. In return, they will show greater recognition, appreciation, and persistent efforts and strengthen their affective commitment to the organization (Chiang and Hsieh, 2012). Career growth in this organizational support climate will more easily translate into affective commitment. According to attribution theory, when knowledge workers realize the help and attention of the enterprise, they will have positive emotions and attribute their career growth to the platform and resources provided by the organization, as well as their professional quality and serious and responsible attitude, and believe that the organization will continue to provide such support in the future (Meyer and Herscovitch, 2001). There is also a significant relationship between organizational support and employee engagement (Al-Omar et al., 2019). A sense of organizational support can negatively regulate the negative emotions and turnover intention caused by conflicts between work and family such that employees feel the help from the organization and are willing to continue to retain the identity of organization members (Giao, 2020). Organizational support can also improve employees’ satisfaction with internal communication, and then affect employee engagement level (Veri, 2021). Accordingly, we propose:

H5: Perceived organizational support moderates the relationship between career growth and affective commitment.

H5a: Perceived organizational support moderates the relationship between career goal progress and affective commitment.

H5b: Perceived organizational support moderates the relationship between professional ability development and affective commitment.

H5c: Perceived organizational support moderates the relationship between organizational rewards development and affective commitment.

**FIGURE 1:**
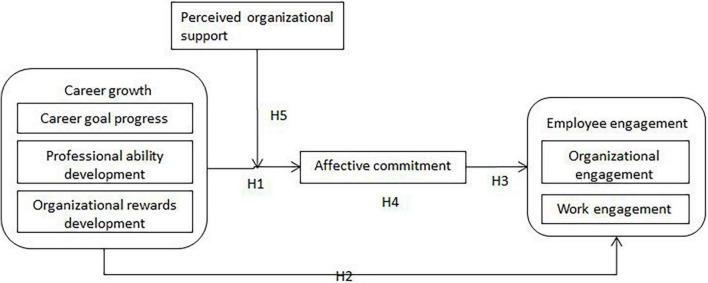
Theoretical model.

Based on the above analysis, the theoretical model of this study is shown in Figure 1.

## Research Design

### Sample and Data Collection

This study collected data through a questionnaire survey. We conducted our survey in April 2020. The final sample consists of both online and offline participants. The offline survey visited some technology-intensive enterprises in the Anhui, Jiangsu, and Zhejiang provinces. We distributed paper questionnaires to enterprises with a relatively large number of knowledge-based employees, such as the aerospace, computer, and biotechnology industries. Online, we distributed the survey to the knowledge workers that the institute could contact through network channels. Most of them were distributed in the Beijing, Shanghai, Anhui, Jiangsu, and Zhejiang provinces, and they worked in state-owned enterprises, foreign companies, private enterprises, and joint ventures. A cover letter attached to the questionnaire assured participants that their participation was voluntary and that their responses were for research purposes only.

A total of 467 questionnaires were sent out, and 396 were received (a response rate of 84.80%). After deleting incomplete and illogical questionnaires, 353 valid questionnaires were obtained. Among them, male knowledge workers accounted for 53.54%, and females accounted for 46.46%. Regarding age, 27.76% of the employees were below 25 years old, 35.41% from 26 to 35 years old, 30.31% from 36 to 45 years old, and 6.51% above 46 years old. Regarding their educational backgrounds, 49.86% of them had a bachelor’s degree, 46.17% had a postgraduate degree, and 3.97% of them held a junior college degree. In terms of working years, 6.23% had less than 2 years, 29.75% had 2–5 years, 46.46% had five to 10 years, and 17.56% had more than 10 years. In category composition, management personnel accounted for 27.76%, technical R&D personnel accounted for 21.53%, marketing personnel accounted for 15.30%, and technical personnel accounted for 35.41%.

### Measurements

The variables in this study were measured using established scales and adapted to the context of the present study. Each questionnaire item corresponding to the constructs was measured using a 7-point Likert scale, anchored from “1-strongly disagree” and “5-strongly agree.” Details are as follows:

(1) Career growth. Referring to the career growth scale developed by Weng and Xi (2013), they were divided into four items of career goal progress, four items of professional ability development, and four items of rewards development, with Cronbach α coefficients of 0.923, 0.904, and 0.917, respectively.

(2) Affective commitment. An investigation was conducted on affective commitment in the three-factor scale of organizational commitment designed by Allen and Meyer (1991). There are six questions in total, and the Cronbach α coefficient is 0.909.

(3) Employee engagement. According to the scale designed by May et al. (2004), there are four items of organizational engagement and five items of work engagement, with Cronbach α coefficients of 0.907 and 0.905, respectively.

(4) Perceived organizational support. Eisenberger et al. (1986) eight-item organizational support scale was used, and the Cronbach α coefficient was 0.918.

According to the existing studies in the occupational field, the gender, education level, and working years of knowledge workers are regarded as control variables.

## Empirical Analysis

### Correlation Analysis

SPSS 22.0 and Mplus 7.4 software were used to analyze the data. Table 1 shows the mean, standard deviation, and correlation coefficient of the six variables.

**TABLE 1:** Statistical test and correlation test.

	Mean	SD	1	2	3	4	5	6
1. Career goal progress	4.615	1.180	1					
2. Professional ability development	4.745	1.090	0.435**	1				
3. Organizational rewards development	4.664	1.178	0.513**	0.472**	1			
4. Affective commitment	4.732	1.050	0.514**	0.537**	0.488**	1		
5. Organizational engagement	4.832	1.064	0.393**	0.468**	0.417**	0.471**	1	
6. Work engagement	4.700	1.105	0.499**	0.439**	0.484**	0.523**	0.463**	1

**p < 0.01.

As displayed, except for the correlation coefficient of career goal progress and organizational engagement (0.393), the correlation coefficients of other variables were all above 0.4, indicating a significant correlation. Career growth and affective commitment were significantly positively correlated with employee engagement, which provided preliminary support for hypothesis testing.

### Confirmatory Factor Analysis and Homologous Variance Test

Harman’s single factor test was conducted. Principal component analysis was used to test the data. The number of factors extracted was 7, and the variance contribution rate of the first factor was 36.713%, lower than Podsakoff’s threshold of 40%. Thus, the common method bias is not a major concern for this study.

**FIGURE 2:**
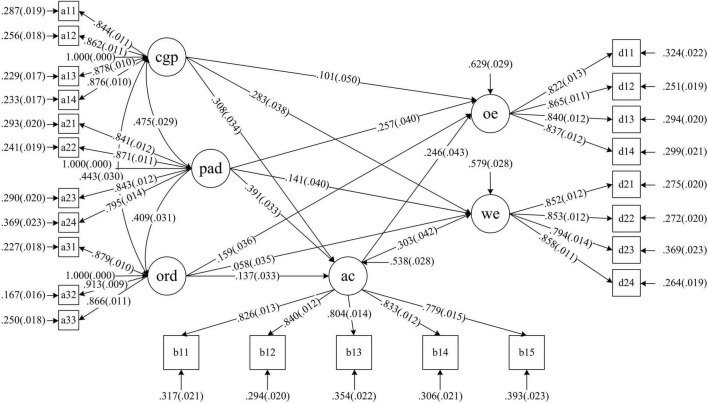
Structural model.

Figure 2 shows the results of the initial model fitting degree test: χ^2^/df = 2.002,SRMR = 0.031 (<0.05), RMSEA = 0.034 (<0.08), CFI = 0.985, and TLI = 0.983, all in line with the corresponding requirements.

### Direct Effect Analysis

As can be seen from Figure 2 and Table 2 on the influence of career growth on employee engagement in the structural model, apart from the standardized path coefficient of organizational rewards development on work engagement (0.058, which failed to reach the level of significance), all others had significant positive effects. Therefore, H1a, H1b, H1c, H1d, and H1e was supported, while H1f has not been verified. H1 is partially formed. Other paths have significant positive effects, and H2a–c, H3a, and 3b have been verified, as well as H2 and H3.

**TABLE 2:** Direct effect analysis.

			Standardized estimates	T	*P*
oe	<—	cgp	0.101	2.560	**
oe	<—	pad	0.257	6.350	***
oe	<—	ord	0.159	4.437	***
we		cgp	0.283	7.524	***
we	<—	pad	0.141	3.557	***
we	<—	ord	0.058	1.649	0.099
ac	<—	cgp	0.308	9.025	***
ac	<—	pad	0.391	11.859	***
ac	<—	ord	0.137	4.106	***
oe	<—	ac	0.246	5.679	***
we	<—	ac	0.308	7.409	***

**p < 0.01, ***p < 0.001. cgp, career goal progress; pad, professional ability development; ord, organizational rewards development; ac, affective commitment; oe, organizational engagement; we = work engagement.

### Mediating Effect Test

The bootstrap method was used to test the mediation effect in Mplus 7.4, and the calculation times were set to 5,000 times to check the path coefficient and significance of the indirect effect.

It can be seen from Table 3 that the path coefficients of career goal progress → affective commitment → organizational engagement are all significant, and the 95% confidence interval of the direct path does not contain 0, indicating a partial mediating effect. Therefore, H4a–e have been established. The path of organizational return growth → affective commitment → job engagement is also significant, and the confidence interval of its direct path contains 0, indicating that it has a complete mediating effect. H4f is established, and therefore, H4 is confirmed to be established.

**TABLE 3:** Mediating effect test.

Effect	Intermediary path	Standard estimate	*P*	Confidence interval	
				Lower 2.5%	Upper 2.5%
Indirect effect	cgp→ac→oe	0.069***	***	0.039	0.111
	cgp→ac→we	0.091	***	0.056	0.137
Direct effect	cgp→oe	0.092	0.029*	0.012	0.180
	cgp→we	0.272	***	0.179	0.367
Total effect	cgp→oe	0.161	***		
	cgp→we	0.362	***		
Indirect effect	pad→ac→oe	0.093	***	0.054	0.147
	pad→ac→we	0.123	***	0.076	0.185
Direct effect	pad→oe	0.249	***	0.150	0.353
	pad→we	0.145	0.005**	0.045	0.248
Total effect	pad→oe	0.342	***		
	pad→we	0.268	***		
Indirect effect	ord→ac→oe	0.029	0.008**	0.012	0.057
	ord→ac→we	0.038	0.006**	0.016	0.073
Direct effect	ord→oe	0.136	***	0.067	0.209
	ord→we	0.052	0.151	−0.021	0.120
Total effect	ord→oe	0.164	***		
	ord→we	0.090	0.013*		

*p < 0.05, **p < 0.01, ***p < 0.001. cgp, career goal progress; pad, professional ability development; ord, organizational rewards development; ac, affective commitment; oe, organizational engagement; we, work engagement.

### Moderating Effect Analysis

This paper tested the moderated mediation effect through Mplus 7.4, as shown in Table 4. First, the interaction between perceived organizational support and career goal progress had a significant positive impact on affective commitment (a3 = 0.122, *p* < 0.001), and affective commitment had a strong positive impact on organizational engagement (b1 = 0.587, *p* < 0.001). The a3 and b1 coefficients were significant. Therefore, H5a was supported. The positive moderating effect of perceived organizational support on career goal progress and affective commitment was established. Similarly, H5b and H5c have been confirmed. Hence, H5 has been verified.

**TABLE 4:** Moderated mediation effect test.

Intermediary path	Estimate	*P*	Confidence interval	
			Lower 2.5%	Upper 2.5%
ac <— cgp(a1)	0.518	***	0.446	0.590
ac <— pos(a2)	0.238	***	0.194	0.281
ac <— cgp*pos(a3)	0.122	***	0.073	0.171
oe <— ac(b1)	0.587	***	0.498	0.675
ac <— cgp(a1)	0.525	***	0.454	0.596
ac <— pos(a2)	0.238	***	0.194	0.281
ac <— cgp*pos(a3)	0.121	***	0.073	0.169
we <— ac(b1)	0.700	***	0.606	0.794
ac <— pad(a1)	0.576	***	0.499	0.653
ac <— pos(a2)	0.201	***	0.157	0.244
ac <— pad*pos(a3)	0.092	***	0.039	0.144
oe <— ac(b1)	0.599	***	0.509	0.689
ac <— pad(a1)	0.572	***	0.495	0.649
ac <— pos(a2)	0.200	***	0.157	0.243
ac <— pad*pos(a3)	0.092	0.001	0.040	0.145
we <— ac(b1)	0.694	***	0.600	0.788
ac <— ord(a1)	0.515	***	0.44	0.59
ac <— pos(a2)	0.258	***	0.213	0.304
ac <— ord*pos(a3)	0.101	***	0.051	0.151
oe <— ac(b1)	0.591	***	0.503	0.68
ac <— cgp(a1)	0.519	***	0.445	0.594
ac <— pos(a2)	0.258	***	0.213	0.303
ac <— cgp*pos(a3)	0.100	***	0.051	0.149
we <— ac(b1)	0.699	***	0.605	0.792

*Means interactive item, ***p < 0.001.

## Discussion

### Research Conclusion

The conclusions are as follows: (1) Career growth has a positive impact on knowledge workers’ organizational engagement. Career goal progress and career ability development in career growth promote job engagement, but the positive impact of organizational rewards development on work engagement has not been verified. It may be that knowledge workers perceive the growth gained from the tests delivered by the organization as trust in them and are therefore more inclined to attribute it to the organization. (2) Career growth has a positive effect on affective commitment, and affective commitment also has a positive effect on employee engagement. (3) Affective commitment plays a mediating role in the effect of career growth on engagement. (4) Perceived organizational support positively moderated the relationship between career growth and affective commitment.

### Theoretical Implications

Our research contributes to the literature in the following ways. First, in the era of knowledge economy, knowledge workers have become the source of enterprise competitive advantage. Given that a large number of studies show that employee engagement reflected in the attitude toward work, team and organization can bring higher benefits to the organization and promote employees to make conscious efforts to meet organizational goals (Mills, 2005), it is no surprise that understanding the factors influencing knowledge workers’ engagement has become particularly critical. This paper enriches the existing literature on knowledge employee engagement by drawing new attention to career growth as an important predictor.

In addition, most studies take career growth and employee engagement as a single dimensional variable. However, the constituent elements of career growth and engagement are multi-dimensional and should be divided into different dimensions for more detailed discussion. This paper subdivides the two variables of career growth and engagement, and better explores what kind of career growth is more needed by knowledge employees and how to better improve employees’ engagement from the perspective of career objectives, salary promotion and ability growth, which provides new insights for the mechanism of knowledge employees’ engagement.

Third, to obtain a better grasp of how knowledge employee engagement is influenced, we combine affective commitment and organizational support into our research model and theoretically and empirically demonstrate the importance of the two in the process of promoting employee engagement. Most studies discuss how to improve and motivate employees from the organizational perspective. However, only from the perspective of employees can we get their true feelings about the organizational attitude. In view of this, this study proposed to explore from the perspective of employees whether their career growth can be better transformed into affective commitment and engagement when employees feel that the organization support for their work has increased. Therefore, this study fills the research gap of the relationship between career growth, affective commitment, organizational support and employee engagement, empirically tested the hierarchical impact of career growth on knowledge employee engagement, complementing existing relevant research in this field that has ignored psychological level attributes as key drivers of consumer trust.

### Practical Implications

The findings of this research may have implications for the management of sharing platforms. First, it is suggested to improve the career growth platform for knowledge workers and take into account the development needs of individuals and organizations. Targeted career discussions at the level of career goal progress can promote a clear development plan so that individual development goals and organizational planning fit together. In terms of professional ability development, the internal staff training system needs to be improved, and the vocational training content should be enriched by dual development plans—management direction and professional direction. Assign challenging tasks to knowledge workers to promote their career growth, provide a platform for them to share experience and insights, and enhance their professional identity. In terms of organizational return growth, it is necessary not only to meet the salary expectations of knowledge workers but also to achieve an efficient allocation of capital. It is also important to engage in regular face-to-face communication to ensure that the position, remuneration, rights, and responsibilities of employees in the organization are equal, willing to give priority to mobility within the organization, to achieve a win-win situation for the organization and employees and to better balance and meet the needs of the organization.

Second, it is suggested to create a caring enterprise atmosphere and exert the positive influence of affective commitment on knowledge workers’ engagement. Knowledge workers pay more attention to the “soft culture” of the enterprise, and the employee-oriented culture and considerate management are more likely to attract their followers. Enterprises need to improve the staff’s team strength and cohesion. At the same time, the enterprise needs to orient the attention of employees to organizational cultures and create an organizational climate on employees’ work and life care. First, setting up an effective communication feedback mechanism and strengthening vertical communication and good relationships with leading members can also become important factors that affect employees’ affective commitment. Second, a certain degree of care should be given to the family life of employees. Knowledge workers tend to undertake more tasks. If there is a sharp conflict between work and family, the career satisfaction of employees is often reduced, resulting in frequent job hopping. When life problems are solved, employees will naturally devote more attention to their work and do their tasks well, enhancing their dependence and loyalty to the organization and thus improving their affective commitment to the organization.

Third, it is suggested to pay attention to the organizational support needs of knowledge workers and provide targeted and diversified ways of employee care. According to the different forms, organizational support can be divided into emotional organizational support (timely meeting the emotional needs of employees) and instrumental organizational support (providing favorable working conditions and facilities). Enterprises should judge which dominant needs different knowledge workers have and then meet them accordingly. At the same time, knowledge workers’ sense of organizational support should be known regularly, universal problems should be solved through institutional adjustment, and different coping strategies should be given to individual problems based on actual personal situations, importance, and other specific factors. Most importantly, all kinds of support provided by enterprise resources should be publicized at the appropriate time to enhance employees’ perception of organizational support and promote it to play its due moderating role.

### Limitations and Future Research

We pay attention to solving possible theoretical and methodological problems. However, there are still some limitations. First, in terms of research content, the variables involved in this paper are limited. Future research can deeply explore more other mediating and moderating factors. For example, for the factor of organizational justice, Saks (2006) selected two interactive rule factors, procedural justice and outcome justice, as the antecedent variables of engagement, which showed that only procedural justice has a significant positive correlation with organizational engagement, while procedural justice and outcome justice have no significant effect on job engagement. Selecting other variables for research will help to fully reveal the internal mechanism in the process of career growth affecting employee engagement, and facilitate enterprises to design a career development mechanism to improve employee engagement from the perspective of career growth. Second, in terms of research methods, considering our research based on survey data, these cross-sectional data can not fully test the actual relationship between variables, and it is difficult to show the dynamic impact between variables (Geng et al., 2021). Future follow-up studies can be more longitudinal to improve the relationship between variables, and use cross-cultural approaches to study the impact mechanism between these variables, further confirming our findings.

## Data Availability Statement

The raw data supporting the conclusions of this article will be made available by the authors, without undue reservation.

## Ethics Statement

Ethical review and approval was not required for the study on human participants in accordance with the local legislation and institutional requirements. Written informed consent for participation was not required for this study in accordance with the national legislation and the institutional requirements. Written informed consent was obtained from the individual(s) for the publication of any potentially identifiable images or data included in this article.

## Author Contributions

ZJ-J: conceptualization, methodology, data curation, software, and writing. SH-M: validation and writing—reviewing and editing. All authors contributed to the article and approved the submitted version.

## Conflict of Interest

The authors declare that the research was conducted in the absence of any commercial or financial relationships that could be construed as a potential conflict of interest.

## Publisher’s Note

All claims expressed in this article are solely those of the authors and do not necessarily represent those of their affiliated organizations, or those of the publisher, the editors and the reviewers. Any product that may be evaluated in this article, or claim that may be made by its manufacturer, is not guaranteed or endorsed by the publisher.
